# Severe diastolic dysfunction as a clue to the cause of stroke: a case report

**DOI:** 10.1093/ehjcr/ytae034

**Published:** 2024-01-30

**Authors:** Maya Maalouf, William J Mandel, Charles Pollick

**Affiliations:** Smidt Heart Institute, Cedars-Sinai Medical Center, Los Angeles, CA 90048, USA; Smidt Heart Institute, Cedars-Sinai Medical Center, Los Angeles, CA 90048, USA; Smidt Heart Institute, Cedars-Sinai Medical Center, Los Angeles, CA 90048, USA

**Keywords:** Restrictive cardiomyopathy, Stroke, Transoesophageal echocardiogram, Cardiac amyloid, Left atrial appendage, Case report

## Abstract

**Background:**

The echocardiographic determination of cardiac causes of stroke focuses on the presence of left ventricular thrombus, valvular vegetations, and patent foramen ovale. Transoesophageal echocardiogram (TEE) is indicated when the transthoracic echocardiogram (TTE) is inconclusive or when there is clinical suspicion of cardiac causes that may have been missed by TTE. The presence of severe diastolic dysfunction on TTE in the absence of any other cardiac abnormality or cardiac history is not usually considered a clue to the cause of stroke.

**Case summary:**

This is a case of a 52-year-old woman who presented with a stroke. Transthoracic echocardiogram was inconclusive for source of embolus. Transoesophageal echocardiogram revealed left atrial appendage (LAA) thrombus and severely hypokinetic LAA, despite the patient being in normal sinus rhythm (NSR). Retrospective analysis of diastolic function on the prior TTE revealed severe restrictive diastolic dysfunction with evidence of elevated left ventricular end-diastolic pressure. While technetium pyrophosphate scan was negative, magnetic resonance imaging was consistent with cardiac amyloid and further testing revealed multiple myeloma as the cause of the amyloid light chain amyloidosis. This case highlights the importance of scrutinizing diastolic function in patients with a source of embolus and careful assessment for LAA thrombus on TEE, despite NSR.

**Discussion:**

We present a patient with stroke with inconclusive TTE findings and eventual diagnosis of restrictive cardiomyopathy secondary to cardiac amyloidosis from an undiagnosed multiple myeloma. Severe restrictive diastolic function on TTE may be a clue to the discovery of LAA thrombus on TEE.

**Learning points**

- Restrictive diastolic function should be considered a clue to the cause of stroke.
- The case report demonstrates the importance of scrutinizing the left atrial appendage for thrombus even in patients with sinus rhythm and no history or testing to suggest atrial fibrillation.

## Introduction

Multiple myeloma, a malignant B cell cancer causing overproduction of abnormal plasma cells, can produce excess immunoglobulins that accumulate in various organs, including the heart. These immunoglobulins are amyloidogenic and have the potential to form amyloid deposits in tissues, which can lead to restrictive cardiomyopathy. Amyloid deposition in the myocardium can cause disruption of normal structure and function, leading to diastolic dysfunction.^1^

We report a case of stroke in a patient with sinus rhythm discovered to have cardiac amyloid and left atrial appendage (LAA) thrombus. The findings in this patient emphasize the importance of being mindful of severe diastolic dysfunction in patients presenting with stroke as a clue to aetiology.

## Case presentation

**Figure 1:**
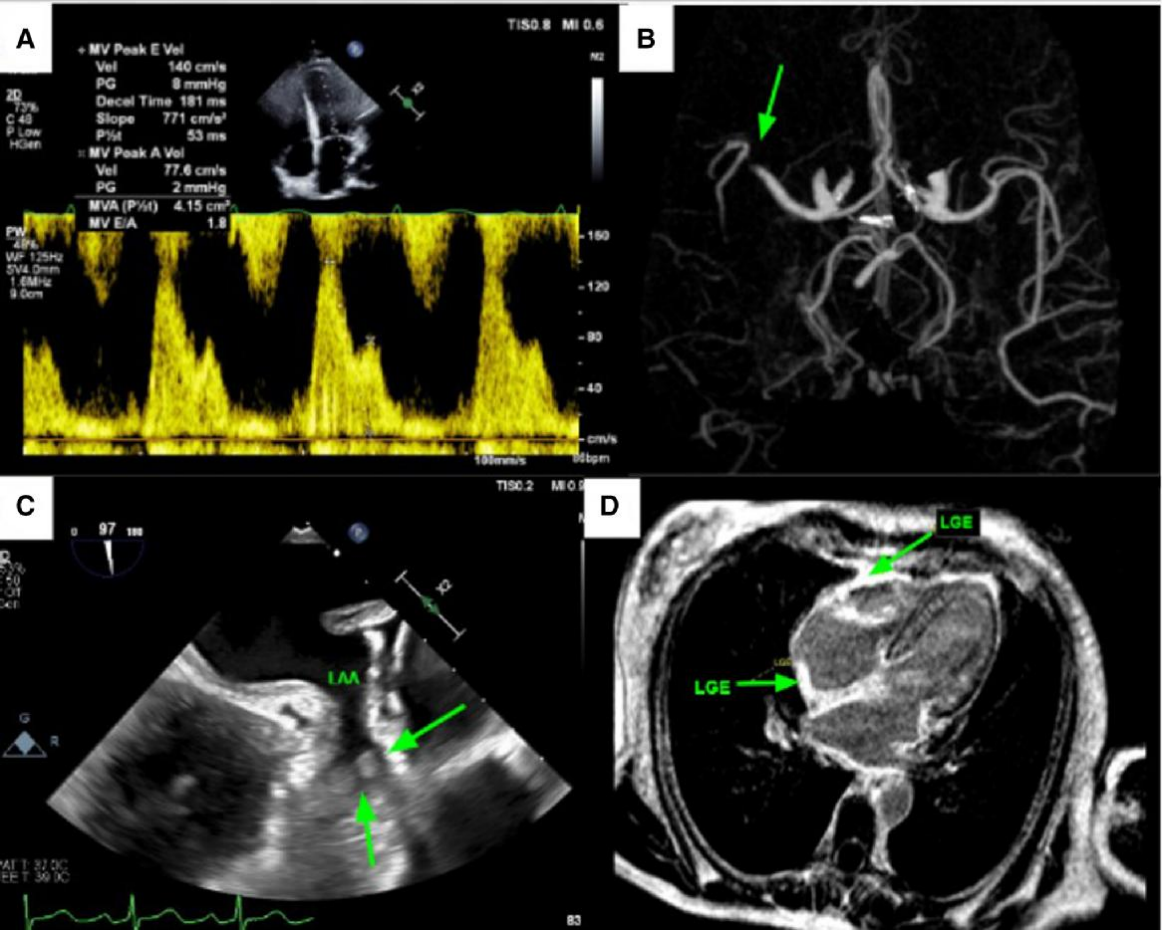
(*A*) Restrictive mitral valve inflow Doppler (transthoracic echocardiogram). (*B*) Computed tomography perfusion brain reveals right middle cerebral artery occlusion indicated by an arrow. (*C*) Transoesophageal echocardiogram reveals left atrial appendage thrombus indicated by arrows. (*D*) Cardiac magnetic resonance imaging reveals diffuse infiltrate and fibrosis indicating cardiac amyloidosis.

**Figure 2:**
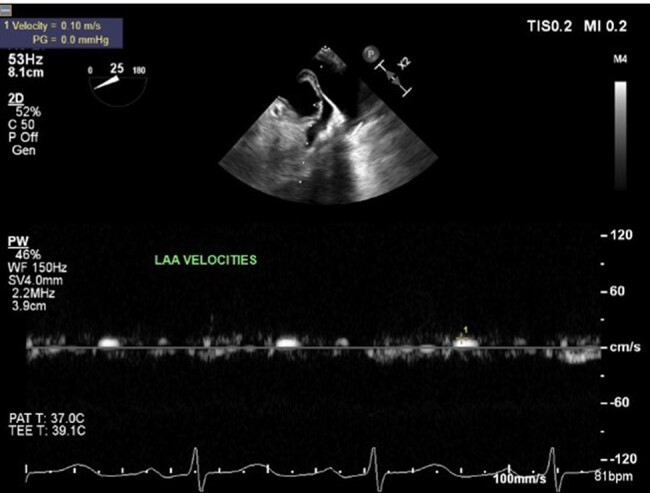
Low left atrial appendage emptying velocities (8 cm/s: *N* 60 ± 14 cm/s) indicating hypokinetic left atrial appendage (pulsed Doppler evaluation during transoesophageal echocardiogram).

**Figure 3:**
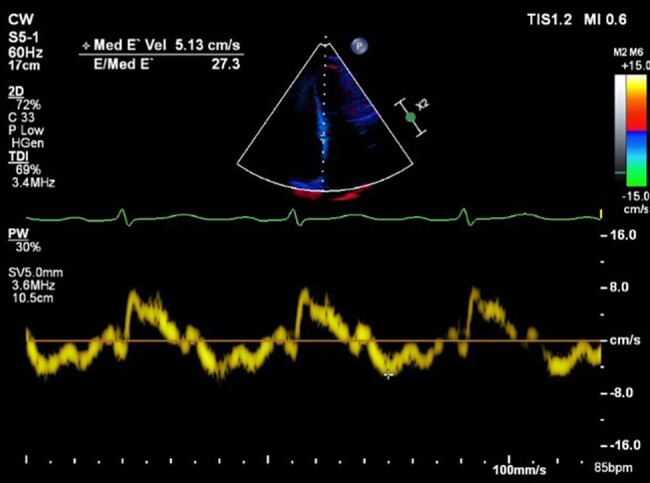
Abnormal medial E/e′ velocities (27.3) (tissue Doppler during transthoracic echocardiogram).

**Figure 4:**
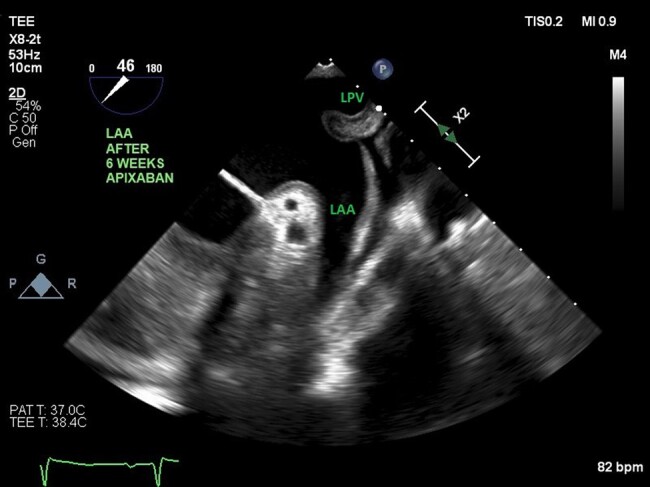
Resolution of left atrial appendage thrombus on apixaban therapy for 6 weeks (transoesophageal echocardiogram of left atrial appendage at 46°). LPV, left pulmonary vein.

**Figure 5:**
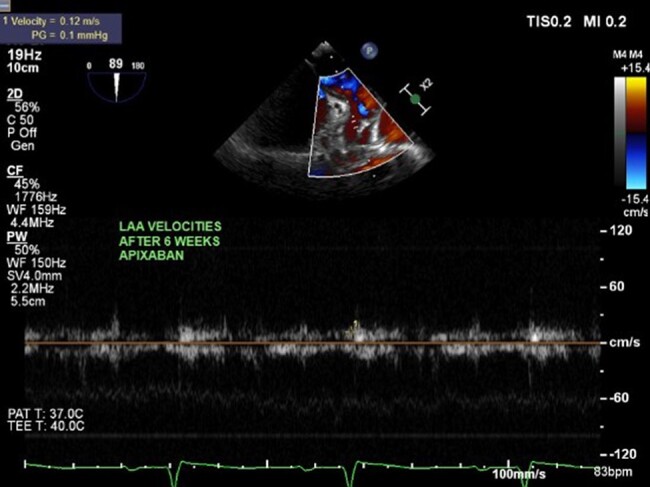
Low emptying left atrial appendage velocities (8 cm/s: *N* 60 ± 14 cm/s) reveals persistent hypokinetic left atrial appendage (pulsed Doppler evaluation of left atrial appendage during transoesophageal echocardiogram).

A 52-year-old woman was admitted with acute left-sided facial droop and left-sided weakness. She had a history of periorbital bruising. Computed tomography workup confirmed an acute middle cerebral artery (MCA) territory infarct (*Figure 1B*). Transthoracic echocardiogram (TTE) showed ejection fraction (EF) 71% and was negative for valve disease, and bubble study was negative for a patent foramen ovale. The patient was discharged home, and 13-day ambulatory electrocardiogram (ECG) recording between the TTE and first transoesophageal echocardiogram (TEE) did not show any episodes of atrial fibrillation. Transoesophageal echocardiogram was subsequently performed to look for a source or route of embolus, and it revealed LAA thrombus (*Figure 1C*) and hypokinetic LAA as evidenced by low LAA velocities (*Figure 2*), despite being in NSR. Transthoracic echocardiogram performed 3 weeks before the TEE revealed Doppler indices (*Figures 1A* and *3* of MV inflow and medial E/e′ velocities) consistent with restrictive physiology: lateral E/e′ 21, medial E/e′ 27.3, and mitral valve (MV) inflow E/A 1.8. Subsequent cardiac magnetic resonance imaging (MRI) revealed evidence of diffuse infiltrate and fibrosis (*Figure 1D*), consistent with cardiac amyloidosis. The patient was referred to a haematologist, and serum and urine electrophoresis confirmed diagnosis of multiple myeloma. The patient was started on apixaban and TEE repeated 6 weeks later which showed resolution of LAA thrombus (*Figure 4*) with persistent poor LAA emptying velocities (*Figure 5*). The patient has since begun and is tolerating a regimen to begin granulocyte colony stimulating factor (G-CSF) collection for auto stem cell transplantation.

## Discussion

Standard workup to determine the aetiology of stroke includes TTE and TEE with saline contrast study to look for patent foramen ovale which may be the route of a paradoxical embolus. In a relatively young woman with no evidence of atrial fibrillation, the left atrial appendage may be overlooked as a cause of stroke as there would be no expectation of this from the history: not only was there no evidence of atrial fibrillation, mitral stenosis, nor systolic dysfunction, but the patient was also in sinus rhythm. It was, therefore, a surprise that we discovered LAA hypokinesis with reduced emptying velocities both of which likely accounted for the detected thrombus.^2^ In retrospect, it became clear that the profound diastolic dysfunction found on the initial TTE was a clue to the presence of possible LAA thrombus as restrictive cardiomyopathy may lead to high left atrial pressure which could predispose to LAA dysfunction and thrombus. The elevated E/e′ ratios suggested elevated left ventricular end-diastolic pressure (LVEDP) and, hence, possible elevated left atrial pressure. In addition, the cardiac MRI showed extensive fibrosis that encompassed the ventricles and left atrium which by itself may have been the reason for poor LAA function and predisposition to thrombus formation.

The vast majority of patients with left atrial appendage thrombus has coexisting atrial fibrillation. Agmon and colleagues^3^ demonstrated that only 1 out of 380 patients with left atrial thrombus had sinus rhythm without high risk factors such as coexisting MV disease or a history of paroxysmal atrial fibrillation. Thrombus usually does not occur in acute atrial fibrillation, when atrial appendage contraction is normal, but becomes increasingly likely with prolonged duration of atrial fibrillation, which may result in a form of LAA cardiomyopathy.^4^ It is speculated that high left atrial pressure may predispose to LAA thrombus formation in the absence of atrial fibrillation.^5^ One prior study demonstrated strong spontaneous contrast (but no thrombus) in a patient with cardiac amyloid and speculated that marked amyloid infiltration may cause atrial dysfunction leading to thrombus formation in the left atrium.^6^ Another prior study reported two patients with cardiac amyloid in whom LAA thrombus was detected—amyloid was diagnosed before the TEE. Our case is different in that the LAA thrombus prompted us to reevaluate the TTE after the TEE and thereby realized that the patient had a restrictive cardiomyopathy which led to a search for a cause and to the diagnosis of cardiac amyloid.

The findings in this case highlight the importance of careful consideration of severe diastolic dysfunction as a clue to the culprit behind the source of embolus due to LAA hypokinesis and LAA thrombus formation.

## Supplementary Material

ytae034_Supplementary_Data

## Data Availability

The data underlying this article are available in the article and in its online Supplementary material.

## Lead author biography

Maya Maalouf is currently a second year medical student at California University of Science and Medicine. She completed her bachelor’s degree at the University of California, Berkeley, and went on to obtain a master’s degree in Global Medicine at Keck School of Medicine.

## Supplementary material

**Consent:** We have written patient consent and confirm compliance with Committee on Publication Ethics (COPE) guidelines.

**Funding:** None declared.

## References

[1] Sharma N , Howlett J. Current state of cardiac amyloidosis. Curr Opin Cardiol 2013;28:242–248.PMID: 23324855. 23324855 10.1097/HCO.0b013e32835dd165

[2] Pollick C , Taylor D. Assessment of left atrial appendage function by transesophageal echocardiography. Implications for the development of thrombus. Circulation 1991;84:223–231. 2060098 10.1161/01.cir.84.1.223

[3] Agmon Y , Khandheria BK, Gentile F, Seward JB. Clinical and echocardiographic characteristics of patients with left atrial thrombus and sinus rhythm. Circulation 2001;105:27–31. 10.1161/hc0102.101776 11772872

[4] Pollick C . Left atrial appendage myopathy: the importance of serial transesophageal assessment in atrial fibrillation. Chest 2000;117:297–298. 10669662 10.1378/chest.117.2.297

[5] Karabay CY , Zehir R, Guler A, Oduncum V, Kalayci A, Aung SM, et al Left atrial deformation parameters predict left atrial appendage function and thrombus in patients with sinus rhythm with suspected cardioembolic stroke. Echocardiography 2013;30:572–581. 23305610 10.1111/echo.12089

[6] Santarone M , Corrado G, Tagliagambe LM, Manzillo GF, Tadeo G, Spata M, et al Atrial thrombosis in cardiac amyloidosis: diagnostic contribution of transesophageal echocardiography. JASE 1999;12:533–536. 10.1016/s0894-7317(99)70091-x 10359926

