# Supplementary figures and images for: Severe diastolic dysfunction as a clue to the cause of stroke: a case report
Source: Eur Heart J Case Rep. 2024 Jan 30;8(2):ytae034. doi: 10.1093/ehjcr/ytae034 (PMC10883693; doi:10.1093/ehjcr/ytae034)

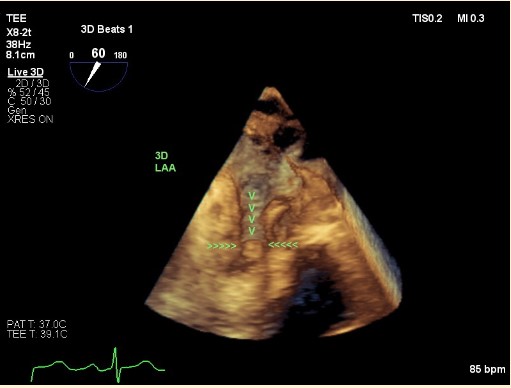

Supplement: ytae034_Supplementary_Data [file ytae034_supplementary_data.zip › EHJ.supplemental.3D LAA.jpg]
